# 4.8-μm CO-filled hollow-core silica fiber light source

**DOI:** 10.1038/s41377-024-01615-x

**Published:** 2024-10-18

**Authors:** Xuanxi Li, Linyong Yang, Zhiyue Zhou, Zhixian Li, Hao Li, Wenxi Pei, Wei Huang, Jing Shi, Luohao Lei, Meng Wang, Zefeng Wang

**Affiliations:** 1 College of Advanced Interdisciplinary Studies, National University of Defense Technology, Changsha, 410073 China; 2 Nanhu Laser Laboratory, National University of Defense Technology, Changsha, 410073 China; 3 Hunan Provincial Key Laboratory of High Energy Laser Technology, National University of Defense Technology, Changsha, 410073 China

**Keywords:** Fibre lasers, Solid-state lasers

## Abstract

Mid-infrared (MIR) fiber lasers are important for a wide range of applications in sensing, spectroscopy, imaging, defense, and security. Some progress has been made in the research of MIR fiber lasers based on soft glass fibers, however, the emission range of rare-earth ions and the robustness of the host materials are still a major challenge for MIR fiber lasers. The large number of gases provide a variety of optical transitions in the MIR band. When combined with recent advances in low-loss hollow-core fiber (HCF), there is a great opportunity for gas-filled fiber lasers to further extend the radiation to the MIR region. Here, a 4.8-μm CO-filled silica-based HCF laser is reported for the first time. This is enabled by an in-house manufactured broadband low-loss HCF with a measured loss of 1.81 dB/m at 4.8 μm. A maximum MIR output power of 46 mW and a tuning range of 180 nm (from 4644 to 4824 nm) are obtained by using an advanced 2.33-μm narrow-linewidth fiber laser. This demonstration represents the longest-wavelength silica-based fiber laser to date, while the absorption loss of bulk silica at 4824 nm is up to 13, 000 dB/m. Further wavelength expansion could be achieved by changing the pump absorption line and optimizing the laser structure.

## Introduction

The extension of the wavelength range of fiber lasers has garnered significant interest owing to its inherent benefits of compactness, stability, and high efficiency in laser emission^1–3^. The mid-infrared (MIR) fiber lasers are particularly noteworthy due to their wide range of applications in various fields, including medicine, industrial processing, communication, and military defense^4^. The increasing significance of MIR lasers can be attributed to their diverse characteristics, which are propelled by the above-mentioned applications^5^.

In the past decades, the utilization of rare-earth-doped fiber lasers has emerged as a highly efficient method for achieving output in the MIR^6–15^. It is well-known that the transmission loss of silica fiber, which is extensively utilized in the near-infrared range, is significantly elevated in the MIR band due to the constraints imposed by phonon energy (up to 1100 cm^−1^)^4^. Therefore, soft glass fibers with low phonon energy are utilized to mitigate the pronounced non-radiative transition between energy levels, thereby facilitating the attainment of MIR output^16^. Nevertheless, the phonon energy associated with mature fluoride fibers (509 cm^−1^) remains excessive for extended wavelengths, thereby imposing a constraint on the output wavelength of fiber lasers, which has historically been confined to 4 μm^17,18^. As a result, there has been a significant emphasis on chalcogenide fibers that possess lower phonon energy (200 cm^−1^), leading to the recent attainment of output wavelengths exceeding 5 μm^19–21^. However, there are still several challenges in realizing MIR chalcogenide fiber lasers^5,16,22^: (i) the toxicity of the raw materials and the relatively poor fiberizability in low loss single mode fibers; (ii) lower rare earth solubility; (iii) severe quenching effect caused by high OH^−^ concentration and (iv) low robustness. Limited by these factors, it is difficult for chalcogenide fiber lasers to obtain stable and efficient output to meet the requirements of advanced MIR fiber sources^5^.

The advent of hollow-core fibers (HCF) has significantly transformed the landscape of fiber lasers operating in the MIR band^23–25^. On the one hand, the HCF maintains the robustness of solid-core silica fiber to a certain extent and exhibits superior thermo-mechanical properties compared to soft glass fiber. On the other hand, the micron-scale core region of HCF offers a favorable platform for the interaction between light and matter. This led to the creation of HCF gas lasers (HCFGL)^26^. More importantly, the HCF exhibits a limited degree of overlapping mode field with the silica material, resulting in significantly reduced losses compared to bulk silica. This exceptional nature makes HCF particularly beneficial in the MIR band^27^.

**Fig. 1:**
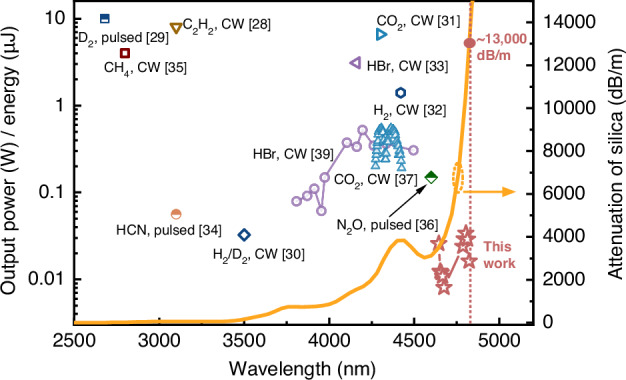
Summary of the state-of-art HCFGLs in terms of output, lasing wavelengths and the material absorption of bulk silica. Continuous wave (CW) and pulsed outputs are both demonstrated^28–37^, with each corresponding to the power and energy on the left *y* axis, respectively. The red star symbols are the results in this work. The absorption loss of the silica material is represented by the yellow curve (right *y* axis) and originates from the Heraeus F300 fused silica glass^43,44^. The vertical dashed line indicates the absorption loss of the silica material at 4824 nm

Taking advantage of HCF, various HCFGLs have achieved efficient operation from 2.68 to 3.5 μm. The loss of silica further increases significantly with the expansion of wavelength. Figure 1 shows a summary of the state-of-art HCFGLs in terms of lasing wavelengths and the material loss of bulk silica^28–37^. The initial achievement of a HCFGL with an output exceeding 4 μm was accomplished through the utilization of stimulated Raman scattering (SRS) of hydrogen^38^. However, as a result of the elevated SRS threshold, the pump system typically manifests as an intricate and costly pulsed light source. Population inversion is an additional operational mechanism employed in HCFGLs. The vibrational energy levels exhibited by gas molecules offer a broad spectrum of radiation, particularly in the wavelength range exceeding 4 μm, which is challenging to attain using rare-earth ions. HCFGLs operating in the MIR range have demonstrated effective generation of high-power, wide-tuned output within the 4-μm wavelength band. This achievement has been realized through the utilization of population inversion in gases such as CO_2_ and HBr^37,39^. In 2019, Aghbolagh et. al reported a 4.6-μm N_2_O-filled HCFGL with an output energy of 150 nJ^36^. This is the silica-based fiber laser with the longest output at present. These works demonstrate the benefits of utilizing HCFGLs for achieving advanced MIR output. Nevertheless, researchers are consistently pursuing the expansion of the wavelength of fiber lasers. In contrast to the above-mentioned gases, carbon monoxide (CO) gas exhibits the ability to generate longer wavelengths spanning from 2.5 to 8.2 μm, accompanied by a notable power output^40^. In the context of conventional optically pumped CO gas lasers, it is possible to achieve an output of 4.8 μm^41^. However, the CO-filled fiber laser has not yet been reported, primarily due to the overly extended wavelength band. While it is true that advanced HCFs have the capability to minimize the overlap between material and mode domains to approximately 10^−5^, the transmission loss of HCFs is still affected by the significant loss of silica materials in the MIR band^42^. According to the curve presented in Fig. 1, it can be observed that there is a significant increase in the loss of silica when the wavelength surpasses 4.6 μm^43,44^. Specifically, the loss reaches a value of 13,000 dB/m at a wavelength of 4.8 μm.

In this work, we represent a nearly 5-μm CO-filled laser based on all-silica fiber for the first time. In order to achieve such a long wavelength laser output, we utilized a broadband low-loss silica-based HCF covering 2~5 μm. Additionally, we have created a single-frequency fiber laser operating at 2.33 μm, which corresponds to the vibrational transition of CO molecules from the *V* = 0 to *V* = 2 vibrational state. A maximum MIR output power of 46 mW was achieved. The maximum tuning range of the MIR spectrum spans from 4644 to 4824 nm, resulting in a wavelength adjustment of 180 nm by manipulating the pump laser. As far as we know, the present output region of this particular laser represents the longest wavelength for silica-based fiber lasers. By making an adjustment to the pump wavelength and the laser structure, it is possible to attain output with an even broader wavelength.

## Results

### The energy level transitions of the CO molecule

**Fig. 2:**
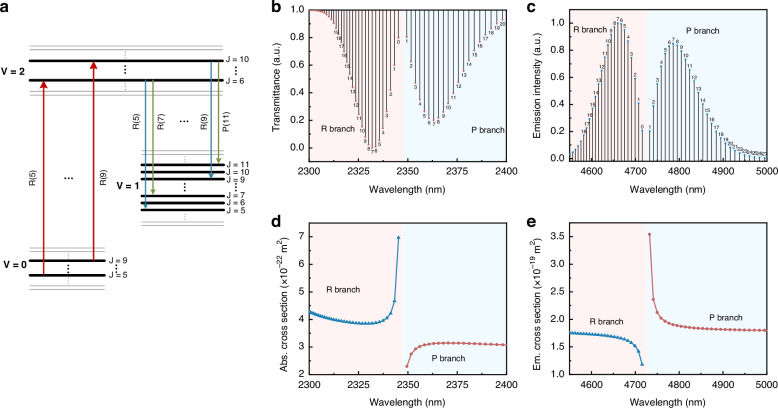
**Energy diagram of CO and corresponding radiative transition properties**. **a** The first overtone absorption transition and the corresponding fundamental band transitions. A comprehensive description of the five absorption lines and ten radiation transition lines utilized in this work is provided. The absorption (**b**) and emission (**c**) spectrum of CO molecules. The absorption cross section (**d**) and emission cross section (**e**) of CO molecules at room temperature and a pressure of 1 mbar

CO has the capability to provide numerous transmission lines within the range of 2.5–8.2 μm, including the fundamental band (*V* + 1 → *V*) and the first-overtone band (*V* + 2 → *V*)^40^. The vibrational quantum number *V* is used to denote the vibrational normal mode of the CO molecule. Historically, there has been extensive research conducted on electric discharge CO lasers, resulting in the achievement of an ultra-high average power output of 200 kW^45^. Hence, considering the advancement of high-power coherent sources within the MIR spectrum, CO lasers present a highly appealing subject. The pump mechanism between the ground state (*V* = 0 vibrational state) and the upper level (*V* = 2 vibrational state) was specifically selected to facilitate the emission on the fundamental band transitions (*V* = 2 → 1), as shown in Fig. 2a. The population residing in the lower vibrational state (*V* = 1) of CO is considered negligible due to the significant energy difference between the *V* = 1 and *V* = 0 vibrational states, which surpasses 2100 cm^−1^. The redistribution of the population from the *V* = 1 vibrational state to the *V* = 0 vibrational state occurs through two main processes: vibrational relaxation (V-V relaxation) and vibrational/translation relaxation (V-T relaxation) between CO molecules^41^. In the case of optically pumped CO lasers, the laser transitions are specifically observed between rotational energy levels that are associated with distinct vibrational states. The rotational quantum number *J* is used to represent the various rotational states within the vibrational level. Based on the quantum mechanical principle of selection, it is observed that radiative transitions occur with a change in the rotational quantum number ∆*J* = +1 (referred to as the R branch, where *J* transitions to *J* + 1) and ∆*J* = − 1 (known as the P branch, where *J* transitions to *J* − 1). The absorption band of CO molecules at 2.3-μm region from *V* = 0 to *V* = 2 state is shown in Fig. 2b^46^. In the thermal equilibrium state, the absorption intensity of the transition lines R(7) and P(7) in CO are strongest due to the densest population in the rotational energy level *J* = 7. In Fig. 2c, the emission intensity of CO molecules transitioning from vibrational level *V* = 2 to *V* = 1 is depicted within the wavelength range of 4.5–5 μm^46^. The distribution of emission lines exhibits similarities to that of absorption lines. It is important to acknowledge that the observed intensity distribution is a result of the non-uniform Boltzmann distribution, and does not accurately reflect the actual cross-sectional profile of the transition. Figure 2d, e display the absorption and emission cross-section curves, respectively. The P-branch emission cross-section typically exhibits greater strength compared to the R branch due to the larger Einstein coefficient^41^. In contrast to the emission situation, it is observed that the absorption cross-section of the R branch is greater in magnitude when compared to that of the P branch. Hence, selecting the R-branch absorption line as the pump wavelength facilitates the attainment of a higher pump absorption efficiency. The corresponding calculation method and parameters are provided in the supplementary file.

### Broadband low-loss HCF

Undoubtedly, HCF is an essential component within HCFGLs. One notable distinction between HCF and solid-core fiber lies in the relatively low overlap between the material and modal fields. This attribute confers significant benefits in regions characterized by substantial material loss, such as the ultraviolet and infrared domains^44^. However, the transmission of HCF continues to be a challenge due to the significant material loss of silica in the MIR range, particularly beyond 4.5 μm. In 2019, Yu et al. demonstrated a HCF with a low loss of 40 dB/km at 4 μm, but the transmission region did not exceed 4.2 μm^44^. Subsequently, Davidson reported that a HCF was accompanied by low losses in the 4.3–5.2 μm region, and the average loss was less than 1 dB/m from 4.5 to 4.7 μm^47^. However, when considering the implementation of HCFGLs, it becomes imperative to consider both the pump band and the laser transmission band. The application potential of such HCF is limited due to its high transmission loss (3 dB/m) in the region of 2–2.5 μm. In a recent study conducted by Newkirk et al., findings were presented regarding a low-loss HCF^48^. The measured loss of this fiber was determined to be 0.316 dB/m at a wavelength of 4.63 μm, and simulations indicated a loss of approximately 0.35 dB/m at a wavelength of 2.3 μm. Regrettably, the transmission region does not surpass a wavelength of 4.7 μm. To cater to the requirements of CO-filled HCFGL, an initial step involved the characterization of a broadband low-loss HCF possessing a transmission range spanning from 2 to 5 μm.

**Fig. 3:**
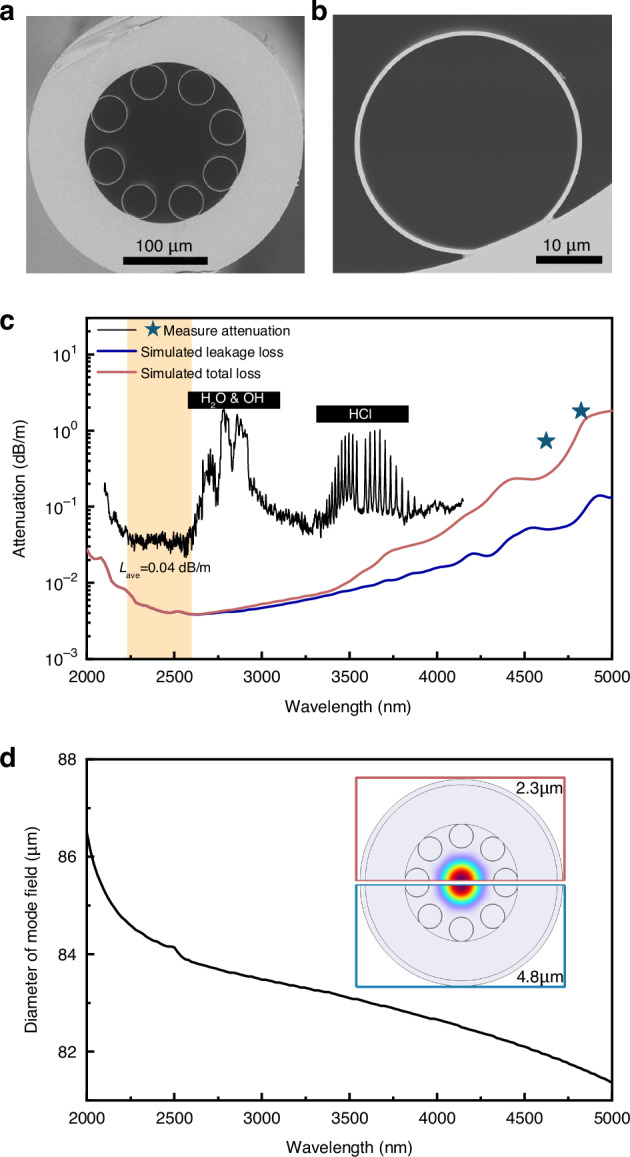
**Characteristics of the in-house manufactured HCF utilized in this work**. **a** SEM images of the cross section of the HCF. **b** Illustrates a partial enlargement. **c** Comparison between simulated and measured loss of the HCF. Losses due to molecular absorption are marked with black areas. **d** Simulated diameter of the LP_01_ mode. Inset shows the simulated intensity profiles of the LP_01_ mode at 2.3 μm and 4.8 μm, respectively

The HCF utilized in this work was produced using the stack-and-draw technique. The scanning electron microscope (SEM) images are presented in Fig. 3a, b. The structure comprises eight non-touching single-ring tubes that form a circular shape around an air core with a diameter measuring 107 μm. During the fiber drawing process, precise control of gas pressure was implemented to ensure the capillary did not collapse and to maintain structural uniformity. The capillary possesses an inner diameter of 39 μm. The working wavelength range for low loss guidance was determined by the thickness of the capillary, as postulated by the anti-resonant reflecting optical waveguide model^49^. To ensure that the absorption and emission band of the CO molecule are situated within the low-loss region, the thickness of the capillary was intentionally set at 900 nm, leading to a first resonance wavelength of approximately 1.87 μm. A decrease in capillary thickness can result in a reduction of modal overlap, thereby mitigating the material loss induced by silica^42,44^.

Figure 3c displays the fiber attenuation spectrum obtained through the implementation of the cutback method. Within the wavelength range of 2.23–2.6 μm, it can be observed that the HCF exhibits a transmission region that remains relatively constant. The average loss, *L*_ave_, within this range was measured to be 0.04 dB/m. This particular wavelength range effectively encompasses all the absorption lines associated with the CO molecule in its *V* = 0 → 2 vibration state, as discussed in Fig. 2. The transmission loss in the vicinity of 2.33 μm was measured to be around 0.05 dB/m, which aligns with the absorption line of the R(5) to R(9). Due to the inherent constraints in supercontinuum source detection, achieving precise loss spectrum measurements beyond the wavelength of 4.15 μm was unattainable. The measurements of cutback using a CO-filled HCFGL, which is described in the following section, demonstrate a loss of 0.73 dB/m at 4644 nm and a loss of 1.81 dB/m at 4824 nm. These results are visually represented by the pentagram in Fig. 3c. In terms of comparison, the attenuation measured in the bulk silica is approximately 3500 dB/m and 13,000 dB/m within the respective wavelength bands mentioned, as discussed in Fig. 1. Since many molecular species exhibit strong absorption resonances in the MIR band, different absorbing regions were found from 2.6 to 4 μm. Absorption lines of H_2_O in the atmosphere and OH in the silica contribute to significant losses around 2.8 μm. A rovibrational absorption band around 3.6 μm was also found, which we attributed to the remaining HCl^44,50^. These absorption lines can be eliminated by purging with nitrogen and will not affect the HCFGL.

A numerical simulation employing finite element analysis was used to extrapolate the loss of the HCF in additional spectral bands^51^. In the MIR spectral band, especially above 3.7 μm, the significant mechanisms that cause attenuation are leakage loss and material absorption of silica^42,44^. Hence, the simulation solely took into account these two variables. The simulation did not account for additional loss mechanisms that contribute to the overall decay of HCF, including surface scattering loss, bending loss, bulk scattering, surface scattering, and molecular gas absorption^42^. The lack of these factors led to the simulation results underestimating the measured losses at 2.3–2.6 μm. At wavelengths of 3.7 μm and beyond, the attenuation calculated in numerical simulations aligns well with experimental measurements.

Increasing the size of the air core can effectively decrease both leakage loss and material loss^42,44^. The utilization of laser delivery is advantageous; however, in the context of laser generation, a larger mode field area results in a diminished energy density, thereby increasing the pumping threshold. Figure 3d presents the diameter of the fundamental mode within the 2 ~ 5 μm range, alongside the simulated intensity distribution of the LP_01_ mode at wavelengths of 2300 nm and 4800 nm, respectively. The mode field diameter exhibits a slight decrease as the wavelength increases. Specifically, at wavelengths of 2300 nm and 4800 nm, the corresponding mode field diameters were calculated to be 84.5 μm and 81.7 μm, respectively.

### 2.33-μm fiber pump source

Historically, there existed two distinct categories of pump sources utilized in the operation of optically pumped CO gas lasers. One option is founded upon the utilization of electric discharged CO lasers, which possess intricate systems and are capable of emitting lasers across multiple spectral bands^52^. Another option is the OPO system, which typically necessitates the use of a complex apparatus capable of generating pulsed output^41^. In contrast, the use of a fiber laser as the pump source will be favorable to the compactness of the overall structure of HCFGL. Recently, there has been a significant focus on Tm^3+^-doped fiber lasers operating within the short-wave MIR range (2~2.5 μm)^53,54^. This attention stems from the compact design of these lasers and the wide emission spectrum exhibited by Tm ions. Moreover, the fiber-based amplifier structure is highly beneficial to achieving a narrow linewidth output^55^.

**Fig. 4:**
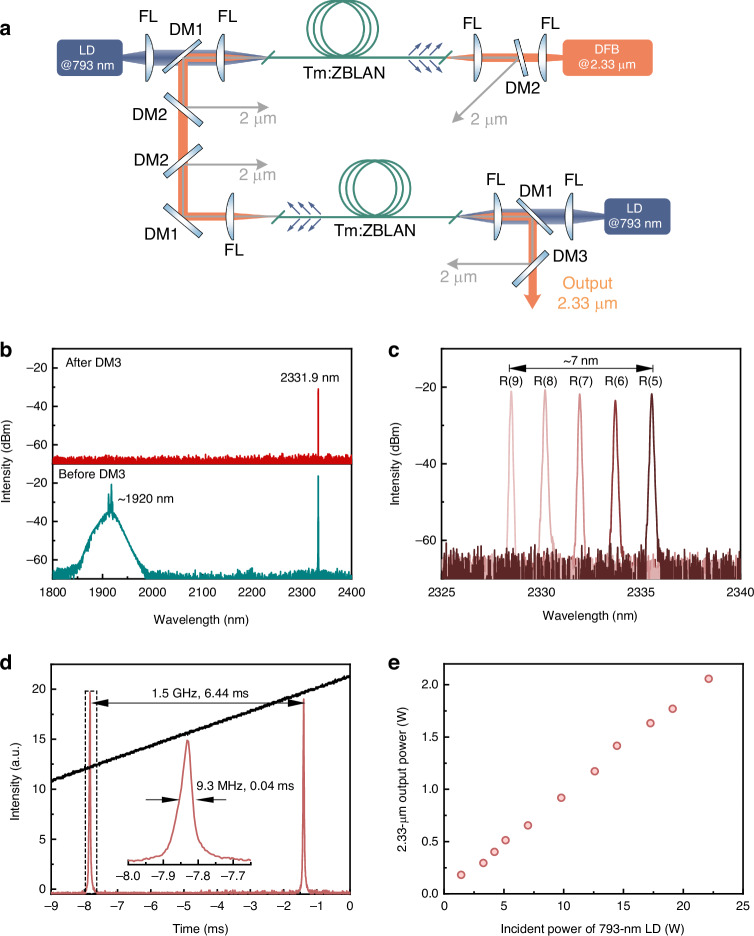
**Characteristics of the 2.33-μm fiber pump source**. **a** Experimental setup of the Tm: ZBLAN fiber amplifier. The system employs an optical free space due to the absence of fiber components specifically engineered for 2.3 μm. FL focus lens. **b** Comparison of output spectra before and after DM3. **c** The measured spectrum of the pump system seeded by chosen absorption line wavelengths. **d** The F-P spectrum of the 2.33-μm laser output. **e** The 2.33-μm output power of Tm fiber laser versus incident pump power at 793 nm. Modifying the wavelength of the output does not result in significant alteration to the power of the 2.33-μm output

A 2.33-μm Tm^3+^-doped fiber amplifier has been developed to acquire a pump source that corresponds to the *V* = 0 → 2 vibrational absorption line of the CO molecule. The laser setup is depicted in Fig. 4a. The overall configuration comprises a two-stage backward pumping amplifier. The single-mode Tm-doped fibers utilized in the amplifier were ground-state pumped by 793-nm laser diodes (LD). It should be noted that the high phonon energy of solid core silica fiber leads to a strong non-radiative relaxation and attenuation above 2.2 µm^5^. Therefore, we selected commercial ZBLAN fiber with low phonon energy to achieve an efficient operation of 2.3 µm. Considering that stable splicing can be achieved between ZBLAN fiber and silica fiber, an all-fiber amplification system operating at 2.3 μm can be realized. In order to achieve a highly precise output with minimal spectral width, the seed laser utilized a single-frequency distributed feedback (DFB) diode laser, which exhibited a standard output wavelength of 2331.9 nm and a linewidth less than 2 MHz.

The utilization of the ground-state pumping technique and the effective cross-relaxation progress in Tm: ZBLAN fiber results in the emergence of a competitive laser transition at a wavelength of 2 μm^54–56^. Hence, the implementation of multiple dichroic mirrors (DM) was employed between amplifier stages and at the final output termination in order to selectively eliminate unwanted laser emissions. A further description of the amplifier structure can be found in the Supplementary File.

The amplified spectrum before and after DM3 for a maximum output power were recorded by an optical spectrum analyzer (OSA), as shown in Fig. 4b. The output after DM3 demonstrated a spectrum with high purity at a wavelength of 2.33 μm, and no notable instances of amplified spontaneous emission (ASE) were detected. By manipulating the operational temperature and current of the DFB laser, it is possible to achieve a precise tuning range of approximately 7 nm. This range effectively encompasses the five absorption lines associated with the CO molecule, specifically from R(5) to R(9), as shown in Fig. 4c. The spectral characteristics were examined in greater detail through the utilization of a scanning Fabry-Perot (F-P) interferometer, which possessed a minimum resolution of 7.5 MHz. The spectra depicted in Fig. 4d illustrate the results obtained upon adjusting the wavelength to 2331.94 nm, which corresponds to the R(7) absorption line of the CO molecule. The observed linewidth at maximum power was measured to be approximately 9.3 MHz, suggesting the absence of obvious broadening phenomenon during the amplification process. For alternative wavelength outputs, the linewidth was maintained at approximately 10 MHz.

Figure 4e illustrates the power output characteristics of the Tm: ZBLAN fiber amplifier. The laser achieved a maximum output power of 2.06 W when subjected to an incident pump power of 22.1 W. As far as we know, this power level represents the highest attainable performance for Tm fiber lasers operating within the 2.3-μm wavelength range. The output power of the 2.3-μm wavelength in Tm fiber was not significantly affected by the tuning of wavelength due to its wide and smooth emission spectrum in the 2.3-μm band^54^. This implies that the system can maintain a consistent output power for the five pump absorption lines of CO molecules.

### CO-filled HCFGL

**Fig. 5:**
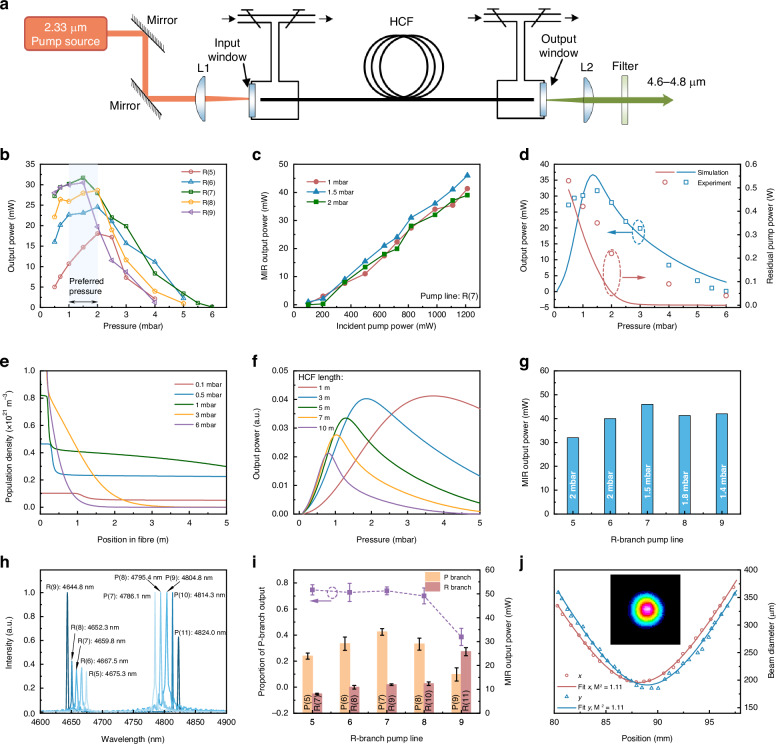
**Characteristics of the CO-filled HCFGL**. **a** The experimental layout for CO-filled HCFGL. **b** For different pump absorption lines, the relationship between MIR output power and pressure. **c** The measured output laser power at different CO gas pressures as a function of the incident pump power when pumped by the R(7) absorption line. **d** The variation of residual pump power and output power with CO pressure under the R(7) pump absorption line, and the pump power is fixed at 0.8 W. **e** The simulated population density distribution of the upper level along the longitudinal direction of the fiber at various pressures. **f** The simulated relationship between the output power, gas pressure, and fiber length. **g** The highest power generated by different pump absorption lines at their respective optimal pressures. **h** The measured output spectra of the CO-filled HCFGL pumped at different absorption lines. **i** For different pump absorption lines, the proportion of the P-branch output to the total output power (left ***y*** axis) and the respective powers of P branch and R branch (right ***y*** axis). **j** Typical output laser beam quality, where the inset shows the beam profile at the waist position

Figure 5a illustrates the experimental configuration of the optically pumped CO-filled HCFGL. Like other HCFGLs, the overall configuration was a typical single-pass structure without an external cavity. The pump laser and CO gas were confined in the HCF to obtain a sufficient gain and generate laser-like output based on ASE. The 2.33-μm pump laser, which was collimated, was free space coupled into the 5-m-long HCF using an uncoated plano-convex lens (L1). In order to adjust and align the pump laser path, two mirrors with a silver-plated surface were inserted prior to L1. The two ends of the HCF were positioned within enclosed gas cells equipped with transparent windows. These cells were connected to a gas pipeline, allowing for the introduction of CO gas. The window employed at the pump end was a CaF_2_ crystal with a 5-mm thickness and a D-coating. Upon careful evaluation of the insertion loss associated with each individual component, it has been determined that the overall coupling efficiency of the pump laser stands at approximately 65%. At the output terminal, the window was an uncoated CaF_2_ crystal. The residual pump laser and MIR laser output were collimated using an uncoated CaF_2_ lens (L2), and subsequently separated by a MIR filter. It should be noted that the use of coated optics in the system will further improve the utilization of the pump laser. The output loss due to uncoated optics has been eliminated in the calculation.

The selection rule influences the spectral outcomes when a specific absorption line of CO molecules is stimulated. This stimulation results in the production of two distinct spectral line outputs, namely the R branch and the P branch. As discussed in Fig. 2, these branches correspond to the quantum mechanical rotational states of ∆*J* = +1 and ∆*J* = − 1, respectively. Therefore, in this paper, the different spectral line outputs of CO lasers are distinguished through the description of the pump absorption line.

The investigation of the impact of changes in CO pressure on various pump lines was conducted in the experiment, as shown in Fig. 5b. Under the incident pump power of 0.8 W, it was observed that the optimal pressure range for the five pump lines, namely R(5) to R(9), fell between 1 and 2 mbar. A reduction or augmentation in pressure will result in a decline in output power. Upon increasing the pressure to 5 mbar, it was observed that the radiation emitted at wavelengths R(5) and R(9) became undetectable. Upon further increasing the pressure to 6 mbar, it was observed that only the absorption line of the R(7) was present. The optimal pressure for CO lasers is comparatively lower than that of CO_2_ and HBr lasers under similar continuous-wave pump conditions^37,39^. This discrepancy primarily arises from the greater transmission loss of the HCF at longer wavelengths (4.6~4.8 μm). It leads to the CO laser necessitating lower pressure to achieve a higher net gain^36^.

Figure 5c illustrates the relationship between the MIR output power and the pump power under various gas pressures when the pump absorption line was R(7). A maximum power output of 46 mW was achieved, corresponding to a pressure of 1.5 mbar. The alteration in pressure results in a marginal reduction in output power, while still exhibiting a linear progression. This suggests that the subsequent power scaling can be attained through the straightforward enhancement of the pump power.

We performed numerical simulations by solving the rate equations to clarify the CO output characteristics. The numerical model and parameters are shown in the supplementary file. Figure 5d shows the variation of residual pump power and output power with gas pressure under the R(7) pump absorption line, and the pump power is fixed at 0.8 W. The good match between the simulation and the experimental results demonstrates the accuracy of the numerical model. Residual pumping decreases gradually as pressure increases, as demonstrated by both experiments and numerical simulations. Low-pressure situations have been found to lower the absorption efficiency of the pump, leading to a reduction in output power. Increasing the pressure will directly enhance the pump absorption rate. However, more pump lasers being absorbed does not mean that the output power will increase accordingly. This increase in pressure also leads to significant vibrational and rotational relaxation, ultimately leading to a reduction in the upper-level lifetime. A more straightforward description is shown in Fig. 5e, e displays the population density distribution of the upper level along the longitudinal direction of the fiber at various pressures. It can be clearly found that the increase in pressure within a certain range effectively populates the density of the upper level. However, further increasing the pressure instead depopulates the density, which is even worse than the low-pressure condition. This distribution is also closely related to the length of the fiber. Note that in Fig. 5e, under the pressure of 3 mbar, the density of the upper level in the first 1 m of the fiber is higher than that of low pressure. This indicates that a shorter HCF is required for high-pressure systems. The relationship between the output power, gas pressure, and fiber length is clearly shown in Fig. 5f. As the fiber becomes shorter, the optimal pressure gradually increases. In addition, compared with long fibers, shorter fibers can achieve higher output power to a certain extent, which is attributed to the reduction of transmission loss.

Figure 5g illustrates the power output of the other four pump lines at their respective optimal pressures. The absorption lines R(5), R(6), R(8), and R(9) exhibited maximum power values of 32 mW, 40 mW, 41 mW, and 43 mW, respectively. These power values were observed at the respective preferred pressures of 2 mbar, 2 mbar, 1.8 mbar, and 1.4 mbar. The power distribution of various pump lines exhibits a notable correspondence with the Boltzmann distribution as discussed in Fig. 2c. The power output curves of these four absorption lines are shown in the supplementary file.

Figure 5h displays the representative output spectra obtained using five pump lines. A total of ten spectral lines were observed. The radiation line with the shortest wavelength, measuring 4644.8 nm, is associated with the quantum transition denoted as R(9). Conversely, the spectral line with the longest wavelength, measuring 4824.0 nm, corresponds to the quantum transition labeled as P(11). Both output spectral lines are associated with the R(9) pump absorption line. In the experiment, it was found that no additional radiation lines were detected when varying CO pressure or power conditions, indicating the absence of significant rotational relaxation. This phenomenon exhibits dissimilarities when compared to CO-filled capillary lasers pumped by OPO system^57^. The rotational relaxation from *J* = 8 to *J* = 5 was successfully achieved in such a laser system under a pressure of 67 mbar. One potential explanation for this disparity could be the inefficient rotational relaxation rate within a low-pressure system^41^.

Figure 5i illustrates the spectral proportions of the P and R branch in the radiation spectrum corresponding to the five pump absorption lines at the highest output power. The dominant component of the P-branch transition was observed in the absorption line of the R(5) to R(8). The reason for this phenomenon is attributed to the typically greater emission cross-section associated with the P-branch transition, as depicted in Fig. 2e. Hence, the P-branch transition will be the initial one to attain the threshold. As the pump power is augmented, the R-branch transition will progressively surpass the P-branch transition and assume dominance in the spectrum^39^. However, in this work, the available pump power proved insufficient to fully saturate the gain of the P-branch transition. Consequently, the P-branch transition remained the prevailing factor even at the highest power level. It is noteworthy that the transition in the R-branch for the R(9) absorption line surpassed that of the P-branch transition. The observed phenomenon can be attributed to a significant disparity in fiber loss between the R(9) and P(11) radiation lines, as discussed in Fig. 3c.

The near-field intensity profile of the CO-filled laser was measured at maximum output power with a CCD camera. As shown in Fig. 5j, the beam intensity was measured at the waist position. Move the position of the CCD camera to obtain the beam profiles at different positions, and the beam quality factor M^2^ was measured to be approximately 1.11 in both the horizontal and vertical directions.

## Discussion

**Fig. 6:**
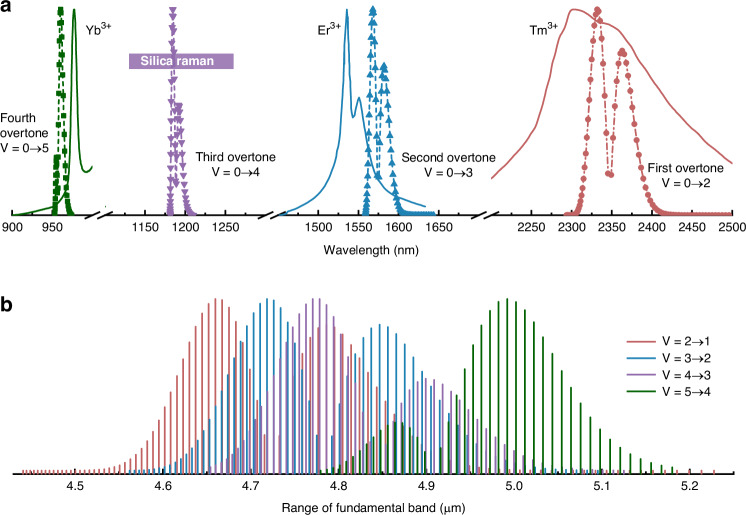
**Wavelength expansion of CO-filled HCFGLs**. **a** The first-order to fourth-order overtone absorption spectra of CO molecules and the emission spectra of Yb, Er and Tm fiber. The wavelength range of silica Raman fiber lasers from 1130 to 1260 nm is calculated based on the first- or second-order Raman frequency shift, as indicated by the purple region. **b** The emission lines associated with the partial fundamental band of CO

As previously mentioned, CO molecules possess a wide range of fundamental bands that enable them to generate infrared output. Figure 2d, e illustrates that CO exhibits comparable absorption and emission cross-sections across various wavelengths, facilitating the expansion of wavelength by a fundamental transition from V = 2 → 1. Figure 6a simultaneously shows the emission spectrum of Tm: ZBLAN fiber in the 2.3-μm band^54^ and the first overtone absorption spectrum of CO molecules^46^, with the aim of investigating the potential of utilizing CO lasers to produce diverse wavelengths. The 2.3-μm band exhibits a wide emission spectrum that spans from 2.3 to 2.5 μm, effectively encompassing all absorption spectral lines associated with the first overtone of CO molecules. Furthermore, it is noteworthy that the highest point of the emission spectrum line in Tm fiber corresponds to the absorption line of the R branch of the CO molecule. This alignment is advantageous for achieving a high rate of pump absorption. In contrast to the 1.8-μm Tm fiber laser or 0.9-μm Yb fiber system, the 2.3-μm fiber laser operates as a quasi-four-level system, thereby enabling efficient short-wave output without being impeded by reabsorption^56,58^. Low-power, widely tunable, and narrow-linewidth 2.3-μm fiber lasers that can be used for CO gas detection have been reported^59,60^. Replacing the DFB seed laser used in Fig. 4a with this laser can serve as the pump source for the CO-filled HCFGL. The low-loss HCF shown in Fig. 3 has low transmission loss in the range of 2.26–2.6 μm and also completely covers the first overtone absorption band. It is crucial to consider the transmission loss of HCF at longer wavelengths. The simulation results indicate that the loss at 5 μm was approximately 2 dB/m, as shown in Fig. 3c. However, thanks to the reduced threshold of HCFGL, it is highly probable that CO-filled HCFGL surpasses the 5 μm output. Using an HCF with lower loss facilitates the extension of the wavelength of CO-filled HCFGL. The nested HCF with multi tubes has lower transmission loss compared to the single-ring HCF. In particular, the nested HCF can effectively reduce bending loss, which is crucial for the miniaturization of HCFGL^61^. Soft-glass-based HCFs have recently emerged, offering a greater bandwidth which is appealing for extending the wavelength of HCFGL^62^. Moreover, unique resonant cavity configurations can greatly decrease the threshold^63^.

Enhanced output spectral lines could be attained by employing alternative fundamental bands. The V = 3 → 2 transition lines of CO molecules in the 4.5–5.2 μm region are also illustrated in Fig. 6b^46^. The emission cross-section for the laser transition from V = 3 → 2 is comparable to that of the transition from V = 2 → 1. Although the absorption cross-section of the second overtone (~10^−24^ m^2^) is smaller than that of the first overtone (~10^−22^ m^2^), a highly developed pump source can effectively compensate for this defect. The second-order overtone absorption lines of CO molecules are illustrated in Fig. 6a^46^. The absorption spectral lines are observed at wavelengths of 1.57 μm. This band possesses the capability to be utilized in conjunction with Er^3+^-doped fiber lasers. These lasers can be realized through fully developed silica fibers^64–66^. The corresponding HCF is also available. By adjusting the wall thickness of the HCF, low-loss transmission in both the pump and the laser band can be achieved. Similar HCFs have been reported in 4.4-μm Raman fiber gas lasers^67^.

Laser transitions from V = 4 → 3 and 5 → 4 are also visible near 5 μm^46^, as depicted in Fig. 6a. The third and fourth overtone absorption bands are located at 1.2 μm and 0.97 μm, respectively, as shown in Fig. 6b. These bands are compatible with advanced Raman fiber lasers and Yb^3+^-doped fiber lasers^68,69^. However, lasers with narrow linewidth that can directly match absorption lines have seldom been reported. The absorption cross-sections of these two bands decreased to 10^−26^ m^2^ and 10^−29^ m^2^, respectively. The decreased absorption cross-section dramatically lowers the likelihood of laser transitions. Thus, there is a significant amount of effort still to accomplish laser transition at V = 4 → 3 and 5 → 4.

In summary, we have demonstrated a MIR CO-filled silica-based fiber laser. By adjusting the pump absorption line, a total of ten laser transitions from 4664 to 4824 nm were observed, which to our knowledge is the longest wavelength silica-based fiber laser. This was achieved by an in-house manufactured broadband low-loss HCF with measured losses of 0.73 dB/km and 1.81 dB/km at 4.64 μm and 4.82 μm respectively. We constructed a 2.33-μm single-frequency fiber laser with high performance to achieve the fundamental band transition of CO molecules. The maximum output power of the MIR laser was 46 mW using the R(7) absorption line under the pressure of 1.5 mbar. Further wavelength expansion could be achieved by changing the pump absorption lines and optimizing the laser structure. We believe that this research will drive the development of new silica fiber laser systems operating at 5 μm and above, which will further enhance new infrared lasers and next generation light sources^70,71^.

## Materials and methods

### The ratio of the P and R branches

The ratio of the output of the P branch and the R branch was distinguished by the spectrum, due to the lack of a filter that can directly separate the two lines. While keeping the measurement conditions unaltered, the spectral information was automatically scanned and recorded by OSA. The respective ratios were then calculated and the uncertainty level was estimated through the intensity of the P branch and R branch lines.

### HCF attenuation measurement

The attenuation of the HCF used in this work was measured using the cutback technique. For the broadband measurement shown by the black curve in Fig. 3c, a MIR supercontinuum source was used as the test source. The source was butt-coupled into the HCF with a bending diameter of 0.5 m. The output of the fiber was connected to the OSA using a bare fiber adaptor. The attenuation was obtained by comparing the spectrum before and after fiber cutback. For the dual-wavelength measurement shown by the pentagram in Fig. 3c, the CO-filled laser with R(9) pump absorption line introduced in this paper was used as the test source. The MIR output light was coupled into the HCF through a 1:1 plano-convex lens system with a bending diameter of 0.8 m to reduce bending loss. The output end of the fiber was also supported by a bare fiber adapter. The power and spectrum at the output of HCF were measured using a power meter and an OSA. The respective power of the R(9) and P(11) output lines can be measured by comparing the spectrum and power. The dual-wavelength attenuation was obtained by comparing the power before and after fiber cutback.

### HCF attenuation simulation

The attenuation was numerically simulated by the finite-element mode method. The geometrical parameters were reconstructed from the SEM image of the HCF, as shown in Fig. 3a, b. The fine mesh size was no more than a fifth of the solved wavelength. The LP_01_ modes were solved from 2000 to 5000 nm in steps of 20 nm. The material loss was calculated using the equations in references^72^. The material absorption of the F300 fused silica was used in the simulation.

## Supplementary information

Supplementary Information for 4.8-μm CO-filled hollow-core silica fiber light source

## Data Availability

The data underlying the results presented in this paper are not publicly available at this time but may be obtained from the authors upon reasonable request.
