# Supplementary material for: 4.8-μm CO-filled hollow-core silica fiber light source
Source: Light Sci Appl. 2024 Oct 18;13:295. doi: 10.1038/s41377-024-01615-x (PMC11487283; doi:10.1038/s41377-024-01615-x)
Supplement: Supplementary file 1 — Supplementary Information for 4.8-μm CO-filled hollow-core silica fiber light source [file 41377_2024_1615_MOESM1_ESM.docx]

Supplementary Information for

4.8-μm CO-filled hollow-core silica fibre light source

Xuanxi Li, ^1,2,^^†^ Linyong Yang, ^1,2,3,†^ Zhiyue Zhou, ^1,2,3,†^ Zhixian Li, ^1,2,3^ Hao Li, ^1,2,3^ Wenxi Pei, ^1,2^ Wei Huang, ^1,2^ Jing Shi, ^1,2^ Luohao Lei, ^1,2^ Meng Wang, ^1,2,3^ and Zefeng Wang ^1,2,3,*^

*^1^College of Advanced Interdisciplinary Studies, National University of Defense Technology, Changsha, 410073, China*

*^2^Nanhu Laser Laboratory, National University of Defense Technology, Changsha, 410073, China*

*^3^Hunan Provincial Key Laboratory of High Energy Laser Technology, National University of Defense Technology, Changsha, 410073, China*

[*zefengwang_nudt@163.com](mailto:*zefengwang_nudt@163.com)

**Table of Contents:**

S1: 2.33-μm Tm: ZBLAN fiber amplifier

S2: The characteristic of CO molecules

S3: The simulation model of CO-filled HCFGL

S4: The mid-infrared laser output power when the pump absorption lines were R(5), R(6), R(8) and R(9)

S1. 2.33-μm Tm: ZBLAN fiber amplifier

A schematic diagram of the two-stage 2.33-μm fiber amplifier is shown in Fig. S1. The experimental setup employed two double-clad Tm: ZBLAN fibers with core and clad diameters measuring 7.5 μm (NA = 0.14) and 120 μm (NA = 0.5), respectively. The cutoff wavelength was approximately 1.9 μm. The fiber was cleaved at an angle of ∼ 9° at both ends to minimize the amount of back-reflected light. The pump power was provided by two commercial 793-nm laser diodes (LD) with a fiber pigtail of 105-μm core diameter (NA = 0.22). The seed laser utilized in the experiment was a single-frequency distributed feedback (DFB) diode laser with a power output of 5 mW and a linewidth of less than 2 MHz. It was equipped with a fiber pigtail that had a core diameter of 7 μm and a NA of 0.2. The standard operating wavelength of the seed laser was measured to be 2331.9 nm.

In the first-stage amplifier, the pump light was coupled into the fiber by means of two uncoated focus lenses with a focal length of 13.5 mm (FL1 and FL2) and a 45° inclined dichroic mirror (DM1) that exhibited high transmission at 793 nm and high reflectivity within the range of 1.8 to 2.4 μm. The DM1 was employed for the purpose of reflecting lasers with wavelengths of 2 and 2.3 μm. The estimated efficiency of the pump coupling was determined to be 70%. The pumping end of fiber was mounted in a water-cooled sink at 18°C and high refractive index glue was covered on the opposite end to prevent thermal damage and filter the residual pump light, respectively. The seed laser was effectively coupled into the fiber through the utilization of FL2, FL3, and DM2. The focal length of FL3, which has been coated with an anti-reflection coating at a wavelength of 2.3 μm, was measured to be 13.5 mm. A DM2 with high transmission at 2.3 μm and high reflectivity at ∼2 μm was intentionally tilted at an angle of 8° between FL2 and FL3. This tilt was implemented with the purpose of isolating the forward laser within the 2-μm wavelength range. The power of the signal that was coupled into the core of the gain fiber was ∼ 2 mW. The extraction of laser output at a wavelength of 2.3 μm from the 2-μm output was achieved through the utilization of the DM2.

The 2.33-μm laser output from the first-stage amplifier was coupled into the second-stage amplifier using the DM1 and FL2. The pump light was coupled into the fiber from the opposing end and the lenses employed were identical to those utilized in the first-stage amplifier. The final 2.33-μm laser output was achieved through the utilization of the DM3. The DM3 and the DM2 have the same parameters, the difference in numerical labels is only for the convenience of distinction.


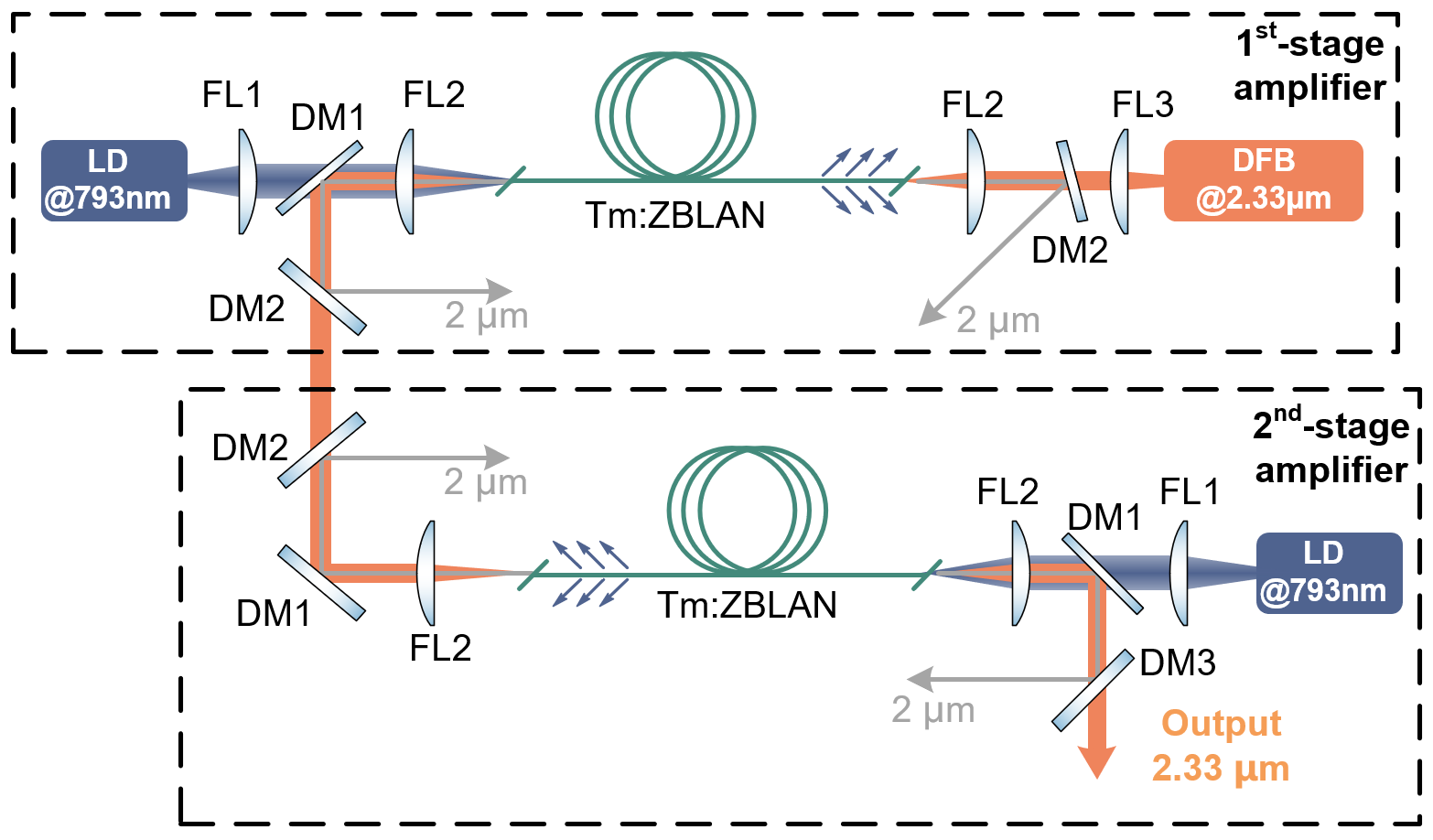


**Figure S1.** Experimental setup of the Tm: ZBLAN fiber amplifier.

S2. The characteristic of CO molecules

a. The absorption linewidth

The absorption linewidth of the R(5) to R(9) absorption lines of CO molecules was measured by scanning the pump wavelength and then measured the power transmitted through the HCF with different pressures, as shown in Fig. S2. Through Voigt line fitting, it is found that for different pump lines, the absorption linewidth of CO molecules was between 290 and 370 MHz. This shows that the homemade 2.33-μm narrow-linewidth (less than 10 MHz) pump system can be fully absorbed by CO molecules.

**Figure S2.** Measured CO absorption linewidth for different absorption lines. **a** R(5) absorption line; **b** R(6) absorption line; **c** R(7) absorption line; **d** R(8) absorption line; **e** R(9) absorption line; **f** Calculated absorption linewidth.

The line shape of CO molecular is mainly affected by collision broadening and Doppler broadening, and ultimately appears as a Voigt line shape function:

$S_{\upsilon}(v)=\frac{\Delta v_{c}}{\text{π}}\sqrt{\frac{m}{2\text{π}k_{B}T}}\int_{-\infty}^{\infty} \frac{\exp\left(-m\upsilon_{z}^{2}/2k_{B}T\right)}{\left(v_{0}+v_{0}\upsilon_{z}/c-v\right)^{2}+\Delta v_{c}^{2}}\text{d}\upsilon_{z}$ (1)

At the center frequency, the Voigt line shape is given by:

$S_{\upsilon}(v_{0})=2\sqrt{\frac{\ln2}{\text{π}}}\frac{\text{erfcx}\left(2\sqrt{\ln2}\Delta\nu_{\text{c}}/\Delta\nu_{\text{d}}\right)}{\Delta\nu_{\text{d}}}$ (2)

where the collision broadening linewidth, Δ*ν*_c_, is equal to *αP*, and *α* is estimated to be 7.0 MHz/torr for CO^1^ and *P* is the gas pressure. ∆*v*_d_ is the linewidth of the Doppler line shape function:

$\Delta\nu_{\text{d}}=\frac{2}{\lambda}\sqrt{\frac{2k_{B}T}{m}\ln2}$ (3)

where *k_B_* is the Boltzmann constant, *T* is the temperature and *m* is the mass of the CO molecule. Figure S2(f) shows the absorption linewidth of CO molecules from R(5) to R(9) absorption lines under different pressures. It can be seen that different absorption lines have similar absorption linewidth, and are similar to the results measured in the experiment.

b. The absorption and emission cross section

The absorption cross section (*σ_ij_*) and emission cross section (*σ_ji_*) of CO molecules can be expressed as:

$\left\{\begin{matrix} \sigma_{ji}=\frac{A_{ji}\lambda_{ji}^{2}S_{\text{υ}}(v)}{8\text{π}} \\ \sigma_{ij}=\frac{2J_{j}+1}{2J_{i}+1}\frac{A_{ij}\lambda_{ij}^{2}S_{\text{υ}}(v)}{8\text{π}} \end{matrix}\right.$ (4)

where *A_ij/ji_* and *J_i/j_* are the Einstein A-coefficient and rotational quantum number of the corresponding energy level, respectively. The A-coefficients for the relevant (2,0) and (2,1) band transitions are presented in Table. S1 and S2^2^.

Table. S1 CO (2,0) band transition wavelength and A-coefficients^2^

| **Transition** | **Wavelength**  **(nm)** | **A-Coeff.**  **(s^-1^)** | **Transition** | **Wavelength**  **(nm)** | **A-Coeff.**  **(s^-1^)** |
| --- | --- | --- | --- | --- | --- |
|  |  |  | R(0) | 2345.305 | 0.3487 |
| P(1) | 2349.504 | 1.0300 | R(1) | 2343.269 | 0.4218 |
| P(2) | 2351.667 | 0.6813 | R(2) | 2341.275 | 0.4554 |
| P(3) | 2353.873 | 0.6084 | R(3) | 2339.323 | 0.4760 |
| P(4) | 2356.121 | 0.5749 | R(4) | 2337.413 | 0.4906 |
| P(5) | 2358.413 | 0.5546 | R(5) | 2335.544 | 0.5020 |
| P(6) | 2360.747 | 0.5402 | R(6) | 2333.717 | 0.5115 |
| P(7) | 2363.125 | 0.5291 | R(7) | 2331.932 | 0.5198 |
| P(8) | 2365.546 | 0.5200 | R(8) | 2330.188 | 0.5273 |
| P(9) | 2368.011 | 0.5121 | R(9) | 2328.485 | 0.5342 |
| P(10) | 2370.519 | 0.5051 | R(10) | 2326.823 | 0.5407 |
| P(11) | 2373.071 | 0.4988 | R(11) | 2325.203 | 0.5468 |
| P(12) | 2375.668 | 0.4929 | R(12) | 2323.623 | 0.5527 |
| P(13) | 2378.308 | 0.4874 | R(13) | 2322.085 | 0.5585 |
| P(14) | 2380.993 | 0.4822 | R(14) | 2320.587 | 0.5641 |
| P(15) | 2383.723 | 0.4772 | R(15) | 2319.13 | 0.5695 |
| P(16) | 2386.498 | 0.4725 | R(16) | 2317.714 | 0.5749 |
| P(17) | 2389.318 | 0.4678 | R(17) | 2316.338 | 0.5803 |
| P(18) | 2392.183 | 0.4634 | R(18) | 2315.003 | 0.5855 |
| P(19) | 2395.093 | 0.459 | R(19) | 2313.708 | 0.5907 |
| P(20) | 2398.05 | 0.4548 | R(20) | 2312.454 | 0.5959 |

Table. S2 CO (2,1) band transition wavelength and A-coefficients^2^

| **Transition** | **Wavelength**  **(nm)** | **A-Coeff.**  **(s^-1^)** | **Transition** | **Wavelength**  **(nm)** | **A-Coeff.**  **(s^-1^)** |
| --- | --- | --- | --- | --- | --- |
|  |  |  | R(0) | 4715.722 | 22.52 |
| P(1) | 4732.65 | 66.830 | R(1) | 4707.42 | 27.18 |
| P(2) | 4741.278 | 44.310 | R(2) | 4699.225 | 29.27 |
| P(3) | 4750.015 | 39.650 | R(3) | 4691.136 | 30.52 |
| P(4) | 4758.863 | 37.540 | R(4) | 4683.154 | 31.38 |
| P(5) | 4767.822 | 36.300 | R(5) | 4675.276 | 32.04 |
| P(6) | 4776.893 | 35.430 | R(6) | 4667.504 | 32.56 |
| P(7) | 4786.077 | 34.770 | R(7) | 4659.835 | 33.01 |
| P(8) | 4795.374 | 34.240 | R(8) | 4652.27 | 33.39 |
| P(9) | 4804.786 | 33.790 | R(9) | 4644.807 | 33.74 |
| P(10) | 4814.312 | 33.400 | R(10) | 4637.447 | 34.05 |
| P(11) | 4823.955 | 33.040 | R(11) | 4630.189 | 34.35 |
| P(12) | 4833.714 | 32.700 | R(12) | 4623.032 | 34.62 |
| P(13) | 4843.591 | 32.400 | R(13) | 4615.976 | 34.89 |
| P(14) | 4853.586 | 32.110 | R(14) | 4609.02 | 35.14 |
| P(15) | 4863.701 | 31.83 | R(15) | 4602.163 | 35.38 |
| P(16) | 4873.935 | 31.57 | R(16) | 4595.406 | 35.61 |
| P(17) | 4884.291 | 31.31 | R(17) | 4588.748 | 35.84 |
| P(18) | 4894.769 | 31.07 | R(18) | 4582.188 | 36.05 |
| P(19) | 4905.37 | 30.82 | R(19) | 4575.726 | 36.27 |
| P(20) | 4916.094 | 30.59 | R(20) | 4569.361 | 36.48 |
| P(21) | 4926.944 | 30.35 | R(21) | 4563.093 | 36.68 |
| P(22) | 4937.919 | 30.12 | R(22) | 4556.922 | 36.88 |
| P(23) | 4949.021 | 29.89 | R(23) | 4550.847 | 37.07 |
| P(24) | 4960.251 | 29.66 | R(24) | 4544.867 | 37.27 |
| P(25) | 4971.61 | 29.45 | R(25) | 4538.983 | 37.46 |
| P(26) | 4983.098 | 29.22 | R(26) | 4533.194 | 37.64 |
| P(27) | 4994.718 | 29.01 | R(27) | 4527.5 | 37.83 |

S3. The simulation model of CO-filled HCFGL

We performed numerical simulations by solving the rate equations to clarify the CO output characteristics^3^. Since no relaxations in the spectral lines were observed, a theoretical model ignoring R–R relaxation was established.

**Figure S4.** Energy level diagram of CO-filled HCFGL.

For the R-branch pump line in the vibrational energy level form V=0→2 and its corresponding laser transition process (including P- and R-branch), the energy level transition diagram shown in Fig. S4 is constructed. The rate equations can be described as follows:

$\left\{\begin{matrix} \frac{\text{d}N_{0}}{\text{d}t}=-W_{02}N_{0}+\frac{N_{1\text{P}}}{\tau_{\text{10P}}}+\frac{N_{\text{1R}}}{\tau_{\text{10R}}}+\left(W_{20}+\frac{1}{\tau_{20}}+A_{20}\right)N_{2} \\ \frac{\text{d}N_{\text{1P}}}{\text{d}t}=\left(W_{21\text{P}}+A_{\text{21P}}\right)N_{2}-\left(W_{12\text{P}}+\frac{1}{\tau_{\text{10P}}}\right)N_{\text{1P}} \\ \frac{\text{d}N_{\text{1R}}}{\text{d}t}=\left(W_{21\text{R}}+A_{\text{21R}}\right)N_{2}-\left(W_{12\text{R}}+\frac{1}{\tau_{\text{10R}}}\right)N_{\text{1R}} \\ N_{2}=N_{\text{t}}-N_{\text{1P}}-N_{\text{1R}} \end{matrix}\right.$ (5)

where *N_i_* represents the population densities of the corresponding energy levels. *τ*_20_=1/(*k*_20_*N*_total_) and *τ*_10P/10R_=1/(*k*_10_*N*_total_) are the non-radiative transition lifetimes of the corresponding energy level. *k*_20_ and *k*_10_ are the rates of non-radiative transition. *A*_20_, *A*_21P_ and *A*_21R_ represent the corresponding spontaneous transition probabilities. *W*_02_ is the R-branch pump absorption rate. *W*_20_ is the stimulated emission rate. In this study, no distinction is made between P-branch and R-branch transitions in this process, and the population densities on *N*_2_ all transition back to *N*_0_. *W*_12P/12R_ and *W*_21P/21R_ are the stimulated absorption and emission rates of the corresponding laser transitions, respectively. These stimulated absorption and emission rates are given by

$\left\{\begin{matrix} W_{02/20}=\frac{\sigma_{\text{02/20}}}{A_{\text{eff}}hv_{\text{02/20}}}P_{\text{p}} \\ W_{21\text{P/12P}}=\frac{\sigma_{21\text{P/12P}}}{A_{\text{eff}}h\nu_{21\text{P/12P}}}P_{\text{sP}} \\ W_{\text{21R/12R}}=\frac{\sigma_{\text{21R/12R}}}{A_{\text{eff}}h\nu_{2\text{1R/12R}}}P_{\text{sR}} \end{matrix}\right.$ (6)

where, *σ*_02/12P/12R_ and *σ*_20/21P/21R_ are the absorption and emission cross sections of the corresponding energy level transitions, respectively. *P*_p_ is the pump power. *P*_sP/sR_ is the P- and R-branch signal power. *ν* is the corresponding optical frequency. *A*_eff_ is the effective area of the HCF. The equations for the pump and signal laser corresponding to the propagation process can be described as follows:

$\left\{\begin{matrix} \frac{\text{d}P_{\text{p}}}{\text{d}z}=\left[\sigma_{20}\left(N_{2}-\frac{f_{2}}{f_{0}}N_{0}\right)-\alpha_{\text{p}}\right]P_{\text{p}}+A_{\text{eff}}\Omega A_{20}h\nu_{20}N_{2} \\ \frac{\text{d}P_{\text{sP}}}{\text{d}z}=\left[\sigma_{\text{21P}}\left(N_{2}-\frac{f_{2}}{f_{\text{1P}}}N_{\text{1P}}\right)-\alpha_{\text{sP}}\right]P_{\text{sP}}+A_{\text{eff}}\Omega A_{\text{21P}}h\nu_{\text{21P}}N_{2} \\ \frac{\text{d}P_{\text{sR}}}{\text{d}z}=\left[\sigma_{\text{21R}}\left(N_{2}-\frac{f_{2}}{f_{\text{1R}}}N_{1\text{R}}\right)-\alpha_{\text{sR}}\right]P_{\text{sR}}+A_{\text{eff}}\Omega A_{\text{21R}}hv_{\text{21R}}N_{2} \end{matrix}\right.$(7)

where, *α* is the transmission loss. Ω is a factor describing only a fraction of spontaneous emission that will transmit along the HCF direction, acting as the initial seed laser. The parameters are listed in Table S1.

**Table S1.** Parameters used in the simulation^1,4^.

| **Parameter** | **Value** | **Unit** | **Parameter** | **Value** | **Unit** |
| --- | --- | --- | --- | --- | --- |
| *ν*_02/20_ | 1.29×10^14^ | Hz | *f*_2_ | 17 | / |
| *ν*_21P/12P_ | 6.24×10^13^ | Hz | *f*_1P_ | 19 | / |
| *ν*_21R/12R_ | 6.43×10^13^ | Hz | *f*_1R_ | 15 | / |
| *A*_21P_ | 33.79 | s^−1^ | *f*_0_ | 15 | / |
| *A*_21R_ | 33.01 | s^−1^ | *α*_p_ | 0.04 | dB∙m^−1^ |
| *A*_20_ | 0.52 | s^−1^ | *α*_sR_ | 0.73 | dB∙m^−1^ |
| *k*_20_ | 2×10^−17^ | m^3^∙mole^−1^∙s^−1^ | *α*_sP_ | 1.81 | dB∙m^−1^ |
| *k*_10_ | 3.11×10^−18^ | m^3^∙mole^−1^∙s^−1^ | *A*_eff_ | 5.2×10^−9^ | m^2^ |
| Ω | 10^−7^ | m^3^∙s^−1^ |  |  |  |

S4. The mid-infrared laser output power when the pump absorption lines were R(5), R(6), R(8) and R(9)

The mid-infrared laser output power characteristics under the R (5), R (6), R (8) and R (9) absorption lines near their respective preferred pressures are shown in Fig. S4.

**Figure S4.** Measured CO output power for different absorption lines. **a** R(5) absorption line; **b** R(6) absorption line; **c** R(8) absorption line; **d** R(9) absorption line.

References

1. McCord, J. E. *et al.* Experimental investigation of an optically pumped mid-infrared carbon monoxide laser. *IEEE Journal of Quantum Electronics* **35**, 1602–1612 (1999).

2. Gordon, I. E. *et al.* The HITRAN2016 molecular spectroscopic database. *Journal of Quantitative Spectroscopy and Radiative Transfer* **203**, 3–69 (2017).

3. Zhou, Z. Y. *et al.* Numerical simulation and observed rotational relaxation in CW and pulsed HBr-filled hollow-core fiber lasers. *Optics Express* **31**, 4739–4750 (2023).

4. HITRAN spectroscopic database. https://hitran.iao.ru/bands/simlaunch?mol=5.
